# Personal attributes of authors and reviewers, social bias and the outcomes of peer review: a case study

**DOI:** 10.12688/f1000research.6012.2

**Published:** 2015-06-10

**Authors:** Richard Walker, Beatriz Barros, Ricardo Conejo, Konrad Neumann, Martin Telefont

**Affiliations:** 1 Frontiers, EPFL Innovation Park, Lausanne, 1015, Switzerland; 2 Department of Computer science, University of Malaga, Malaga, 29071, Spain; 3 Institute of Medical Biometrics and Clinical Epidemiology, Charité, Universitätsmedizin, Berlin, 10117, Germany; 4 Blue Brain Project, EPFL ENT CBS BBP / HBP, Geneva, 1211, Switzerland

**Keywords:** Peer review, bias, nationality, gender, language, prestige, random intercept model, authors, reviewers

## Abstract

Peer review is the "gold standard" for evaluating journal and conference papers, research proposals, on-going projects and university departments. However, it is widely believed that current systems are expensive, conservative and prone to various forms of bias. One form of bias identified in the literature is “social bias” linked to the personal attributes of authors and reviewers. To quantify the importance of this form of bias in modern peer review, we analyze three datasets providing information on the attributes of authors and reviewers and review outcomes: one from Frontiers - an open access publishing house with a novel interactive review process, and two from Spanish and international computer science conferences, which use traditional peer review. We use a random intercept model in which review outcome is the dependent variable, author and reviewer attributes are the independent variables and bias is defined by the interaction between author and reviewer attributes. We find no evidence of bias in terms of gender, or the language or prestige of author and reviewer institutions in any of the three datasets, but some weak evidence of regional bias in all three. Reviewer gender and the language and prestige of reviewer institutions appear to have little effect on review outcomes, but author gender, and the characteristics of author institutions have moderate to large effects. The methodology used cannot determine whether these are due to objective differences in scientific merit or entrenched biases shared by all reviewers.

## Introduction

Peer review is the “gold standard” for the evaluation of journal and conference papers, research proposals, on-going projects and university departments and there is a strong consensus in the scientific community that it improves the quality of scientific publications ^1, 2^. As reported by Armstrong, “journal peer review is commonly believed to reduce the number of errors in published work, to serve readers as a signal of quality and to provide a fair way to allocate journal space” ^3^. Surveys of authors and expert reviewers ^4– 6^ support this view. However, many members of the scientific community also believe that peer review is expensive, conservative and prone to bias ^2, 7– 18^. Critics point to the major delays it introduces into the publication process ^17, 19^, to biases against particular categories of papers (e.g. studies challenging conventional wisdom ^20^; replication studies ^21, 22^ and studies reporting negative results ^12, 23^), to the unreliability of the review process ^23– 25^, to its inability to detect errors and fraud ^26^, and to unethical practices by editors and reviewers ^27, 28^.

Another common criticism of peer review is that review results are not determined exclusively by scientific merit, but also by the social and demographic characteristics of authors and reviewers. In some cases, these effects may constitute a form of social bias. For instance, reviewers may give different scores to papers of equal merit by authors with different personal characteristics (e.g. gender, geographical origin, language, institutional affiliation). Differences may also be determined by the interaction between author and reviewer characteristics, i.e. by biases of reviewers with specific attributes for or against particular categories of author. Finally, reviewers with different personal characteristics could score the same paper differently. Although this would not constitute bias, it would mean that the make-up of the review panel for a paper would affect its score, independently of its scientific merit – an undesirable result.

Studies of the effect of potential bias against authors with specific characteristics have focused on gender. For example, 29 reports that the introduction of double blind review in the journal *Behavioral Ecology* led to an increase in the number of accepted papers with female first authors, compared to five similar journals where reviewers were not blinded to author gender. In the same spirit, a widely cited study of grant awards in Sweden ^30^ suggests that proposals from male candidates receive systematically higher evaluations than those from female candidates with similar academic records, a result confirmed by a recent follow-up study ^15^. A meta-analysis of 21 studies of peer review to assess research applications or applications for post-graduate fellowships also found robust gender effects on peer review results ^31^. All these findings suggest that gender bias is real. However, other studies reached opposite conclusions. For instance, Budden’s study of *Behavioral Ecology* was later contested by 32, who found no significant difference between journals that adopted double blind and single blind review. Similarly, a study by Braisher and colleagues suggested that publication success in *Nature* and *Science* is unrelated to gender ^33^.

Studies of potential bias with respect to other author characteristics (e.g. bias for or against authors from particular geographical areas, language bias, bias in favor of authors from high prestige institutions) have been less frequent but have also produced contrasting results, comprehensively reviewed by Lee and colleagues ^34^. A study by Tregenza reports that review results vary with the country of origin of the author ^35^. Marsh and colleagues ^36^, show that grant applications from authors in high-ranking Australian universities are accepted more frequently than applications from authors in lower-ranked institutions. However, the authors of these studies agree that these differences do not in themselves constitute proof of bias and could simply reflect differences in scientific merit, as discussed in 31.

More convincingly, for the purposes of this paper, Peters and Ceci ^37^ report a quasi-experiment demonstrating that papers by authors from high-prestige institutions have a significantly higher chance of acceptance than similar papers by authors with less prestigious affiliations, and participants in surveys of authors are reported to believe in such an effect ^38^. This finding is supported by a study of papers submitted to scientific sessions at the American Heart Association’s annual research meeting ^39^, which shows, not only that mean review scores for papers by authors from institutions in non-English-speaking countries and from institutions of low academic prestige, are lower than those for papers by authors from English speaking countries, and from higher prestige institutions but also that these differences are lower in reviews where the reviewers have been blinded to authors’ identities and affiliations. This is evidence that review scores may indeed be affected by social bias.

Additional evidence can be obtained by studying the *interaction* between author and reviewer characteristics. For instance, an experimental study by ** Lloyd ^40^, reports that manuscripts with female author names have a far higher acceptance rate when they are reviewed by female rather than male reviewers (62% vs. 21%) and that female reviewers accept papers with male author names less frequently than male authors (62% vs. 10%). Link has shown that while US and non-US authors both give higher scores to US than to non-US authors, the difference is significantly higher when the reviewer comes from the USA ^41^. Similarly, Jayasinghe and colleagues report that papers by Australian authors are more likely to be accepted by Australian reviewers than by reviewers from other countries ^42^. Again however, not all the evidence points in the same direction. For instance, Marsh and colleagues’ previously cited study of Australian review practices ^36^ finds no interaction between researcher and reviewer gender.

Compared to evidence for social bias with respect to author characteristics and interaction effects, support for systematic differences in scoring behaviour by reviewers with different characteristics is relatively weak. An experimental study by Gilbert and colleagues finds that the distribution of review scores given by male reviewers were broader (i.e. more extreme) than the distribution of scores from female reviewers ^43^. However, the sample size was small and the effect was observed only for “statistics reviewers” and not for “content reviewers”. Marsh has shown that US reviewers asked to review Australian grant applications give higher review scores than reviewers from other countries ^36^. A study by Wing and colleagues reports that female reviewers were less likely than men to accept or accept with minor revisions, producing a higher proportion of reviews that external assessors judged to be very good or exceptionally good ^44^. By contrast, a study by Caelleigh and colleagues shows no significant effect of reviewer gender ^45^.

Attempts to remedy potential biases present in traditional peer review have led to a diversification of peer review practices, for instance through the use of author-blind and non-selective review, the removal of traditional reviewer anonymity, and the introduction of various forms of community review. To date, however, there have been few attempts to measure their effectiveness. Furthermore, many past studies of bias in peer review are relatively old and it is not clear whether or how far biases detected in the past have been affected by changes in social attitudes. In response to these concerns, we analyze data for the peer review systems used in Frontiers (an open access publishing house which uses a novel interactive review process), three computer science conferences (CAEPIA2003, JITEL2007, and SINTICE07) held in Spain between 2003 and 2007 and four international computer science conferences (AH2002, AIED2003, ICALP2002 and UMAP2011), held between 2002 and 2011.

### Frontiers

Frontiers is a large open access scientific publisher, which published its first journal in 2007. In January 2015, Frontiers had a portfolio of 49 open-access journals with over 50,000 researchers serving on its editorial boards and more than 380 academic specialty sections. Papers are published within these sections. Each paper is assigned to a scientist acting as the editor who coordinates the review process, and is responsible for publication/rejection decisions. Reviewers are selected automatically, based on the match between their individual specialties, and key words in articles submitted for review (or can also be assigned manually by the editor). During the review process, reviewers remain anonymous. However, accepted papers carry the names of their reviewers. This gives reviewers a strong incentive not to accept papers before they have reached a good level of quality.

The Frontier’s review system is designed not so much to *select* papers as to *improve* the quality of papers in a collaborative, interactive dialog between authors (whose identities and affiliations are available to reviewers) and reviewers (who at this stage of the process are anonymous). In other words, Frontiers adopts a single blind review process. At the beginning of the review process, in which the reviewers review submitted papers independently of each other, they are asked to answer a series of open questions concerning different aspects of the paper (see Table 1). The precise set of questions depends on the nature of the paper (original research, review paper, etc.). Authors answer reviewer questions through an interactive forum. This possibility reduces misunderstandings and can significantly accelerate the submission process.

**Table 1.** Sample of questions to reviewers used in the Frontiers review process.

1) Are there any objective errors in the results? If yes, please specify.
2) Is the work ethical in your opinion?
3) Was the research carried out in accordance with established animal use practices?
4) Is any use of human subjects performed according to acceptable standards?
5) Has the clinical trial been registered in a public trials registry?
6) Should an accession number of nucleotide/amino acid sequences be included?
7) Should an accession number for microarray data be included?
8) Does the article describe experiments using a select agent or toxin?

**Figure 1.**
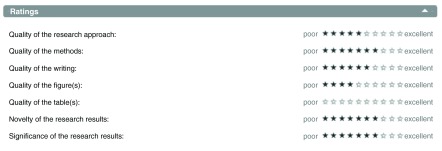
Summary scales used in the Frontiers review system.

In the initial, non-interactive phase of the review process, reviewers can express their overall evaluation of the paper on a range of numerical scales (see Figure 1). However, the use of these scales is not mandatory. Nowhere in the process do papers receive an aggregate numerical score. The final decision to accept or reject a paper is taken by the journal editor, based on the overall results of the interactive review process. Acceptance rates are high. Of the papers in the Frontiers database that had reached a final publication/rejection decision on the date when we extracted the data, 91.5% were published and only 8.5% were rejected.

### IEEE (Spain) and IEEE (international)

The conferences in these two datasets all used WebConf ( http://WebConf.iaia.lcc.uma.es), a computerized system for managing the submission and review of conference papers. WebConf was developed by a team from Malaga University led by one of the authors (RC). WebConf implements a classical review process similar to the processes used by Springer, Elsevier and other large commercial publishers. Conference contributions are usually reviewed by three independent reviewers, occasionally by two or four. Reviewers are chosen by the conference program chair, who draws on a database of potential experts called the program committee. In general, the program committee is made up of authors who have previously submitted papers in a particular area of research and have expressed their willingness to act as reviewers. The WebConf system suggests a list of potential reviewers based on the degree of matching between paper topics and reviewers field of expertise. The final selection is based on the judgment of the program chair.

Reviewers express their opinion of a paper in a conference-specific review form in which they assign scores to the paper on a number of separate scales, covering key areas of evaluation (typically including soundness, originality, clarity etc.) and textual comments. Scores on individual scales are usually expressed in terms of categories (typically: poor, fair, good, excellent). The final publication decision depends on the program chair. If one of the reviewers expresses a strongly negative view of a paper, it will typically be rejected. In cases where there is a very significant difference in reviewers’ opinions, the program chair can ask for an additional review. Acceptance rates vary between a minimum of 29.3% and a maximum of 87%. The combined acceptance rate for the seven conferences in the WebConf database was 57.9%.

## Methods

### Data

**Frontiers.** The Frontiers database includes details of all authors and reviewers for all scientific papers submitted to Frontiers (N=8,565) between June 25, 2007 and March 19, 2012, the name of the journal to which the paper was submitted, the article type (review, original research etc.), the name and institutional affiliations of the authors and reviewers of specific papers, individual reviewers scores for the summary scales shown in Figure 1, and the overall review result (accepted/rejected). At the time of the analysis, 2,926 papers had not completed the review process and were excluded. In another 1,089 cases, reviewers had not assigned numerical scores to the paper, which could not therefore be considered. Our final analysis used 9,618 reviews, for 4,549 papers. Most of the papers in the database come from the life sciences. The majority of authors and reviewers come from Western Europe and Northern America. However, the database contains a substantial number of authors and reviewers from other parts of the world.

**Spanish computer science conferences (IEEE-Spain).** This dataset includes details of 1,131 reviews referring to 411 papers submitted to three IEEE conferences (CAEPIA2003, SINTICE2007 and JTEL2007). The majority of authors and reviewers for these papers come from institutions in Spain and Portugal. The data provided include the name of the conference to which the contribution was submitted, the type of contribution (poster, short paper, full paper etc.) the name, gender and institutional affiliations of the authors and reviewers of specific contributions, individual reviewers scores and the final decision (accepted/rejected). All the papers in the database are in the area of computer science.

**International computer science conferences (IEEE-International).** This dataset provides data for 2,194 reviews, referring to 793 computer science papers submitted to four IEEE conferences (AH2002, AIED2003, ICALP2002 and UMAP2011), managed using WebConf and involving authors and reviewers from all over the world. This dataset provided the same data collected for IEEE-Spain.

### Normalization of author and reviewer names

Names of authors and reviewers were canonized: accent and symbols were removed; double spaces replaced by single spaces, and upper-case characters replaced with lower-case characters. Names were rewritten in the normalized form <first name, last name>. Intermediate names were omitted. The only role of names in our analysis was to allow identification of author and reviewer gender in the case of the Frontiers dataset. Any errors would thus have only a limited effect on the analysis of gender bias for this dataset. Quality controls on automated gender assignment are described below.

### Normalization of institution names

Names of institutions (universities, research institutions, companies) were canonized as above. After normalization, the name of the institution was recoded using the first three words in the full name. The only use of names of institutions in the analysis was to infer their Shanghai ranking. Quality controls on the attribution of Shanghai ranking are described below.

### Gender assignment

Neither the Frontiers nor the WebConf databases included data for author and reviewer gender. In the Frontiers case, gender was inferred semi-automatically in a multistep process. First we matched the first names contained in our database to an open source dictionary providing genders for more than 40,000 first names used in different countries (gender-1.0.0.tgz, downloadable from http://pecl.php.net/package/gender). We then used volunteers of different nationalities (Chinese, Egyptian, Indian, Japanese, Korean, Turkish) to assign genders to first names not contained in the dictionary. Additional names were assigned by manually searching for specific authors and reviewers on Google and Facebook. At the end of this process, we were able to assign genders to 96.4% of authors and 87.1% of reviewers. The majority of unassigned names were Asian (mainly Chinese). Genders for WebConf authors and reviewers were inferred manually, with no missing values in the dataset.

To test the reliability of the two procedures and the impact of possible errors, we randomly selected 125 authors and 125 reviewers for each data set, searched the web sites of their respective institutions to find their genders, and compared them against those generated through our automatic process. For the Frontiers dataset, the analysis showed an error rate of 7.5% mostly due to assignment of female gender to authors and reviewers who were actually male. The error rate for the IEEE Spanish and the IEEE international datasets were much lower (0.0% and 5.2% respectively). The difference was probably due to the broader range of countries (and “unusual” first names) in the Frontiers dataset. For reasons we will consider in the discussion, errors in gender assignment are unlikely to have influenced the conclusions of our study.

### Assignment of countries/geographical area/English vs. non-English speaking

To analyze potential country, regional and language biases, we assigned each author and reviewer to the country of the institution to which they were affiliated, as listed in the original datasets. Authors and reviewers with multiple affiliations were assigned to the country of the first affiliation listed. In the Frontiers dataset, we deduced country information from the affiliation given by authors and reviewers. Since 9.0% of reviews lacked information on first author affiliation and 5.3% lacked the information for reviewers, it was not possible to deduce the geographical location or the language of their affiliated institutions. In the IEEE (Spain) and the IEEE (International) datasets, authors and reviewers provided country data information directly and the number of missing values was <1.0% in both cases. The geographical region and language of authors’ and reviewers affiliated institutions were determined using the country information. USA, UK, Scotland, N. Ireland, Ireland, Australia, Canada, and New Zealand were classified as English-speaking countries. All other countries were classed as non-English speaking.

### University rankings

Authors’ and reviewers’ affiliated institutions were classified in terms of their position in the 2012 Shanghai academic ranking of world universities for the life sciences ( http://www.shanghairanking.com/FieldLIFE2012.html) (Frontiers) and for computer sciences ( http://www.shanghairanking.com/SubjectCS2012.html) (WEBCONF). The institution names in the Shanghai ranking were normalized using the same procedure used to normalize institution names in the three datasets (see above). To check the quality of our automatic assignment of university ranking, we extracted a random sample of 125 universities from each dataset and checked the university ranking manually, finding error rates of 11.2% for Frontiers, and 9.6% for IEEE (International). The IEEE (Spain) dataset was excluded from the analysis for other reasons (see below).

### Calculation of review scores

**Figure 2.**
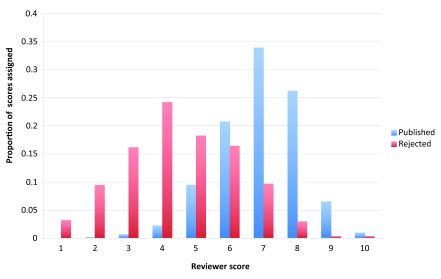
Distribution of reviewer scores for published and rejected papers in the Frontiers dataset.

***Frontiers.*** The Frontiers review process produces a very low rejection rate. This means that the database used for our study contained relatively few rejected papers (N=478). To create a more informative indicator of reviewers’ evaluations, we computed for each paper the average of the scores expressed by each individual reviewer for each of the summary scales shown in Figure 1 (one mean score for each reviewer). A comparison between the distributions of scores for rejected and published papers (see Figure 2) clearly demonstrates the validity of the indicator. However, it should be noted that the indicator is a construction of the authors and played no role in the review process.

**WebConf.** The WebConf system asks each reviewer to assign an overall score to the paper he/she has just reviewed. Scores are expressed on a scale of 0 to 10.

### Statistical analysis

For the purposes of the study, we define bias as the interaction terms *δ _ij_* in the random intercept model:

*y = b + µ _ij_ + β _ij_A _i_ + γ _ij_R _j_ + δ _ij_A _i_R _j_ + ε*          (1)

where y denotes the score given in the review, *b* denotes the random intercept, *i* indexes properties of authors, *j* indexes properties of reviewers, and *ε* is the error term. This method is similar but not identical to the method proposed in 46.

Given a factor *F*, such as *region,* the variables *A _i_* and *R _j_* are indicator (dummy) variables indicating that the first author and reviewer belong to categories i and j of factor F, respectively, that is:

IF one author belongs to category *i* of F, *A _i_=1,* ELSE *A _i_=0*        (2)

AND

IF one reviewer belongs to category *j* of F, *R _j_=1,* ELSE *R _j_=0*        (3)

Thus *β _ij_* is the fixed effect of author category, *γ _ij_* is the fixed effect of reviewer category and *δ _ij_* is the fixed effect of the interaction between author and reviewer category. Since the expected value of the random intercept *b* is 0, the fixed effects allow us to estimate the following mean scores:

*G _ij_ = µ _ij_ + β _ij_ + γ _ij_ + δ _ij_*    author in category *i*, reviewer in category *j*

*G _i $\overset{ˇ}{j}$_* = *µ _ij_ + β _ij_*             author in category *i,* reviewer *not in* category *j*

*G _$\overset{ˇ}{i}$__j_* = *µ _$\overset{ˇ}{i}$__j_* + *γ _ij_*              author not in category *i*, reviewer in category *j*

*G _$\overset{ˇ}{i}$$\overset{ˇ}{j}$_ = µ _ij_*             author *not* in category *i,* reviewer *not* in category *j.*

We define bias *B _ij_* of reviewers from category *j* of factor F towards authors from category i by the expression:

*B _ij_ =* ( *G _ij_* – *G _i $\overset{ˇ}{j}$_*) – ( *G _$\overset{ˇ}{i}$__j_* – *G _$\overset{ˇ}{i}$_*_$\overset{ˇ}{j}$_) *= δ _ij_*       (4)

Since the intercept and main effects cancel out, bias is the interaction term *δ _ij_* and does not depend on the main effects *β _ij_* and *γ _ij_*. In other words, it is independent of any general tendency of authors in category i to write better papers than other authors, or of any tendency of reviewers in category to give generally higher scores. In this setting, reviewers from category *j* are *biased in favor of* authors from category *i,* if B _ij_ > 0 and are biased *against* authors from category *i* if B _ij_ < 0. Bias is significant at a level *α*, if we can reject the null hypothesis:

*H*_0_ : *B _ij_* = 0

Otherwise, we assume absence of bias.

The majority of papers in our databases had multiple authors. In preliminary studies, we explored statistical models that used this data in different ways: (i) the model used only the properties of the first author, (ii) the model used only the properties of the last author, (iii) the model considered the properties of all the authors. The three approaches yielded similar results (data not shown). In what follows, we apply the first method, unless otherwise stated.

### Example

To illustrate the concept of bias defined in ( 4), consider a factor with two levels such as gender. Let *i* and *j* denote female (F), and $\overset{ˇ}{i}$ and $\overset{ˇ}{j}$ male (M). Then the terms *G _ij_ = G _FF_, G _i $\overset{ˇ}{j}$_ = G _FM_, G _$\overset{ˇ}{i}$j_ = G _MF_,* and *G _$\overset{ˇ}{i}$$\overset{ˇ}{j}$_ = G _MM_*, have the following meanings:

*G _FF_*   : mean score when first author and reviewer are female

*G _FM_*  : mean score when first author is female and reviewer is male

*G _MF_*  : mean score when first author is male and reviewer is female

*G _MM_* : mean score when first author and reviewer are male

If we assumed that papers by female authors have the same quality as papers by male authors,

*G _FF_ – G _FM_* > 0       (5)

would imply that female reviewers are biased in favor of female authors and

*G _MF_ – G _MM_* > 0      (6)

would imply that female reviewers are biased in favor of male authors.

However, we cannot make this assumption. We therefore conclude that female reviewers are biased for or against female authors (B _FF_ ≠ 0), only if ( 5) and ( 6), yield different results. If both are equal and positive, we conclude that females give higher scores than men regardless of the gender of the author. If female reviewers have a positive bias, this always implies a negative bias on the part of male reviewers and vice versa. By construction, this method cannot detect biases shared by all reviewers (e.g. a bias against female authors, or authors from a particular geographical region, shared by all reviewers, regardless of gender or geographical origin).

It should also be noted that, in our example, if B = (G _FF_ – G _FM_) – (G _MF_ – G _MM_) > 0, the following statements are equivalent:

- Female reviewers are biased in favor of female authors
- Female reviewers are biased against male authors
- Male reviewers are biased against female authors
- Male reviewers are biased in favor of male authors

Similarly, if

- Female reviewers are biased against female authors
- Female reviewers are biased in favor of male authors
- Male reviewers are biased in favor of female authors
- Male reviewers are biased against male authors

Given the low number of rejections in our datasets, it was not possible to measure author, reviewer and interaction effects on the acceptance and rejection of papers but only on review scores. Effect sizes were measured using Cohen’s d, which provides a standardized measure of the differences between two means. It is generally believed that effect sizes of 0.2 or smaller are small, that effect sizes of around 0.5 are medium and that effects >=0.8 are large. However, the practical impact of an effect of a given size depends on the size of the reference population. Issues related to the practical impact of our findings will be addressed in the discussion.

## Results

### Gender

The automatic gender assignment program described earlier assigned genders to first authors and reviewers for 8,024 reviews from Frontiers, 1,131 from IEEE (Spain) and 2,194 from IEEE (International). The relative proportions of male first authors and reviewers (male authors: 71.9% – 75.0%; male reviewers: 75.1% – 79.3%) were similar in all three datasets. In the Frontiers and IEEE (International) datasets, means scores for male first authors were significantly higher than those for female first authors (Frontiers: difference=0.07, p=0.034, d=0.1; IEEE International: difference=0.28, p=0.001, d=0.2). The IEEE (Spain) dataset showed the same pattern but the difference was not significant (difference=0.40, p=0.29, d=0.3). Reviewer gender had no significant effect on review scores in any of the datasets, nor did the interaction between author and reviewer gender have a significant effect in any of the datasets. In brief, none of the datasets showed evidence of gender bias. The significance of these results will be examined in the discussion. Complete data for the analysis can be found in Data Files 1–4.

### Region

The analysis examined the role of the region of first author and reviewer institutions in determining review scores, and tested for possible regional bias. Authors and reviewers were grouped into 11 geographical regions (Africa, Australia/New Zealand, Central America/Caribbean, Central Asia, Eastern Asia, Eastern Europe, Middle East/North Africa, South America, Southern Asia, Southern Europe, and Western Europe) according to the location of their respective institutions. To avoid problems with the convergence of the mixed model algorithm and to guarantee the statistical power of the analysis, pairs of first author/reviewer regions with less than 30 reviews were discarded. Distributions of author and reviewer regions differed significantly among the three datasets. In the Frontiers and International IEEE datasets, the majority of authors and reviewers came from institutions in North America and Western Europe, while the majority of authors and reviewers in the Spanish IEEE dataset came from institutions in Southern Europe.

In all three datasets, differences between the scores of first authors from different regions were statistically significant, even after application of the Bonferroni correction for multiple hypothesis testing (see Table 2). In the Frontiers dataset, authors from North America scored significantly higher than authors from all other regions and authors from Eastern Europe, Southern Asia, and Southern Europe scored significantly lower. In the IEEE (Spain) dataset, authors from Southern Europe scored significantly higher than authors from other regions. In the IEEE (international dataset), authors from North America again scored significantly higher than authors from other regions, and authors from Southern Europe scored lower. Effect sizes were small to moderate (Cohen’s d between 0.1 and 0.7).

**Table 2.** Differences in mean scores for first authors from institutions in different geographical regions with respect to all other regions. Tables shows all data with uncorrected p<=0.05. * shows significant bias after application of the Bonferroni correction for multiple hypothesis testing (Frontiers *α* = 0.005; IEEE-Spain *α* < 0.0125; IEEE-International *α* < 0.0028).

Frontiers		Bonferroni corrected alpha=0.005			
	N	Mean score region	Mean score other regions	p-value	Cohen's d
*Eastern Asia*	501	7.20	7.40	0.009	0.2
*Eastern Europe*	82	6.85	7.40	0.003*	0.4
*North America*	3,733	7.50	7.32	<0.001*	0.1
*Southern Asia*	86	6.71	7.40	<0.001*	0.5
*Southern Europe*	705	7.21	7.41	0.003*	0.1
**IEEE Spain**		**Bonferroni corrected alpha=0.0125**			
*Central America*	40	5.37	6.11	0.014	0.6
*Eastern Asia*	49	5.57	6.11	0.046	0.4
*South America*	36	5.37	6.11	0.017	0.6
*Southern Europe*	976	6.20	5.39	<0.001*	0.7
**IEEE (International)**		**Bonferroni corrected alpha=0.006**			
*Eastern Asia*	161	5.22	5.67	0.039	0.4
*North America*	503	6.11	5.50	<0.001*	0.5
*Southern Europe*	441	5.29	5.72	0.004*	0.4

Differences in scores linked to the geographical location of reviewer institutions were rare (see Table 3). In the Frontiers dataset, South American reviewers gave scores that were significantly higher than those given by reviewers from other regions, even after application of the Bonferroni correction. In the IEEE (Spain) dataset, there were no significant differences in the scores given by reviewers from institutions in different regions (not shown). In the IEEE (International) dataset reviewers from Australia/NZ gave scores that were significantly higher than the average for reviewers from other regions. In all cases, effect sizes were small to moderate (Cohen’s d in the range 0.1–0.5). The number of reviews whose scores may have been affected was small (1.7% of reviews for Frontiers, none for IEEE-Spain, 6.1% for IEEE-International). 

To test for bias, we applied the random intercept model to all author/review region pairs with more than 30 reviews (see Table 4). After application of the Bonferroni correction, the Frontiers dataset showed no evidence of interaction between author and reviewer region and the other two datasets showed only very limited evidence (IEEE – Spain: strong bias of reviewers from S. Europe in favor of authors from E. Asia; IEEE – International: strong bias of North American reviewers in favor of authors from Eastern Asia). However, the proportion of reviews potentially affected by these biases was small (None for Frontiers, 4.1% of papers for IEEE-Spain, 1.4% for IEEE-International). None of the datasets showed evidence for regional biases previously reported in the literature (e.g. bias of North American reviewers in favor of North American authors; bias of Australian reviewers in favour of Australian authors). We conclude that regional biases, while sometimes large, are idiosyncratic to particular review systems, and probably have only a limited effect on review results. Full data for the analysis can be found in Data Files 5–8.

**Table 3.** Differences in mean scores for reviewers from institutions in different geographical regions with respect to all other regions. Tables shows data with uncorrected p<=0.05. * shows significant bias after application of the Bonferroni correction for multiple hypothesis testing (Frontiers α = 0.005; IEEE-Spain *α* < 0.0125; IEEE-International *α* < 0.0028).

Frontiers		Bonferroni corrected alpha=0.005			
	N	Mean score region	Mean score other regions	p-value	Cohen's d
*South America*	166	7.76	7.39	0.001*	0.3
**IEEE (International)**		**Bonferroni corrected alpha=0.005**			
*Australia/NZ*	135	6.08	5.63	<0.001*	0.4
*Southern Asia*	40	6.21	5.65	0.015	0.5
*Western Europe*	935	5.57	5.73	0.009	0.1

**Table 4.** Bias in mean scores for specific author-reviewer region pairs with uncorrected p-values <=0.05; * shows significant bias after application of the Bonferroni correction for multiple hypothesis testing (Frontiers *α* = 0.001; IEEE-Spain *α* < 0.0125; IEEE-International *α* < 0.0028).

Frontiers		Bonferroni corrected alpha=0.0016			
Author	Reviewer	N	Difference	p-Value	Cohen’s d
E. Asia	E. Asia	130	0.35	0.047	0.3
E. Asia	S. Europe	34	-0.58	0.028	0.4
N. America	E. Europe	36	-0.63	0.034	0.5
**IEEE Spain**		**Bonferroni corrected alpha=0.0125**			
**Author**	**Reviewer**	**N**	**Bias**	**p-Value**	**Cohen’s d**
E. Asia	S. Europe	46	2.34	0.005*	1.9
**IEEE International**		**Bonferroni corrected alpha=0.0028**			
**Author**	**Reviewer**	**N**	**Bias**	**p-Value**	**Cohen’s d**
E. Asia	N. America	31	1.06	<0.001*	0.9
E. Asia	W. Europe	60	-0.46	0.048	0.4
N. America	W. Europe	223	-0.29	0.04	0.2
S. Europe	S. Europe	109	0.40	0.023	0.3
W. Europe	S. Europe	153	-0.30	0.050	0.3
W. Europe	W. Europe	344	0.28	0.026	0.2

### Language

We hypothesized that reviewers could be biased against papers written by authors who were not native English speakers. We, therefore, analyzed potential reviewer bias for and against papers whose first authors came or did not come from institutions in English-speaking countries. As a further test, we analyzed potential bias for and against papers, which had, or did not have, at least one author belonging to an institution in an English-speaking country.

The Frontiers and the IEEE (International) datasets both included large numbers of authors and reviewers from institutions in native English-speaking, and from non-English-speaking countries. In contrast, approximately 97% of the authors and reviewers in the IEEE (Spain) dataset came from Spain. Since none of the papers with an English-language first author, and only one paper with at least one English author, were reviewed by an English-language reviewer, it was not possible to measure bias using the random intercept model. This dataset was therefore discarded from the subsequent analysis.

In the remaining datasets, papers with a first author from an institution in a non-English-speaking country scored significantly lower that papers with first authors from institutions in an English speaking country, regardless of whether they were reviewed by native English-speaking or non-native English speaking reviewers (Frontiers: difference=-0.25, p<0.001, d=0.2; IEEE International: difference=-0.54, p<0.001, d=0.5). Reviewer language had no significant effect on score (Frontiers: difference=0.03, p=0.35; d=0.0); IEEE International: difference=0.01; p=0.80, d=0.0). In neither case did we find a significant interaction between author and reviewer language (not shown). Results for papers with at least one author from an institution in an English-speaking country were similar. Details of the analysis are shown in Data Files 9–11.

### Ranking of author and reviewer institutions

Reviewers from institutions with high academic prestige could be biased in favor of authors from other high prestige institutions and against authors from lower ranking institutions. To test this possibility, we classified all authors and reviewers in the three datasets by the position of their institutions in the Shanghai classifications, as described earlier. The Frontiers and the IEEE International datasets both contained significant numbers of authors and reviewers from universities in all three categories. However, a large majority of authors and reviewers in the IEEE Spain dataset came from universities in category 3. Given the lack of data for authors and reviewers from higher-ranking institutions, this dataset was excluded from the subsequent analysis.

In the Frontiers dataset, authors from universities in category 1 scored significantly higher than authors from category 2 (difference=0.17, p=0.010, d=0.1) and from category 3 (difference=0.22, p<0.001, d=0.2), regardless of the origin of the reviewer. No significant difference was observed between the scores of authors in category 2 and 3. In the IEEE International dataset, authors from universities in categories 1 and 2 received similar scores and both scored significantly higher than authors in category 3 (category 1: difference in scores=0.74, p=<0.001, d=0.6; category 2: difference in scores=0.66, p=<0.001, d=0.6).

In the Frontiers dataset, there was no significant difference between the scores given by reviewers in different categories. The IEEE (International) data showed no significant differences between scores from reviewers affiliated to institutions in category 1 and category 2 or 3 but a significant difference between scores from reviewers in category 2 and 3 (difference in scores=0.20, p=0.008, d=0.2). Neither dataset showed a significant interaction between the prestige of author and reviewer institutions. Full details of the analysis are given in Data Files 12–14.

## Discussion

The results of the study (see Table 5) show that the scores received by papers in peer review depend strongly on the characteristics of the first author (gender, geographical location, language and prestige of the author’s institution). In summary, male authors receive higher scores than female authors, authors from some geographical regions receive higher scores than authors from others; authors from institutions in English-speaking countries receive higher scores than authors in non-English-speaking countries; authors from high prestige institutions receive higher scores than authors from lower-prestige institutions. In several cases effect sizes were relatively large (Cohen’s d in the range 0.5–0.7). In contrast, we find little evidence that scores are affected by the personal characteristics of reviewers, no significant interactions between author and reviewer gender, language, and institutional prestige and only sporadic interactions between author and reviewer region. In brief, the study provides little evidence for social bias, in the sense in which it is defined in our study (see below).

**Table 5.** Summary of main findings.

Finding	Frontiers	IEEE (Spain)	IEEE (International)
Male first authors achieve higher scores than female first authors	X		X
There is no significant difference between scores from male and female reviewers	X	X	X
There is no evidence of gender bias (significant interaction between author and reviewer gender)	X	X	X
In a small number of cases, first authors from particular geographical regions score significantly higher or significantly lower than authors from all other regions.	X	X	X
There are no significant differences between scores given by reviewers from different regions	X	X	X
There is little or no regional bias (little or no evidence for interaction between author and review region)	X	X	X
Authors from institutions in English-speaking countries score higher than authors in non-English speaking countries	X	N/A	X
There is no significance difference between scores from reviewers from institutions in English and non-English speaking countries	X	N/A	X
There is no significant language bias (no interaction between language of author and reviewer institutions)	X	N/A	X
Scores for authors from institutions with high Shanghai rankings are significantly higher than scores for authors from lower ranking institutions	X	N/A	X
There is no significant difference between scores from reviewers from institutions in different Shanghai categories	X	N/A	X
There is no bias in terms of the Shanghai category of author and reviewer institutions (no significant interaction between them)	X	N/A	X

The finding that author characteristics have a significant effect on review scores is compatible with two distinct explanatory hypotheses. The first is that papers submitted by authors with a particular characteristic (e.g. authors from institutions in a particular region) are, on average, of higher scientific merit than papers by authors with different characteristics (e.g. authors from institutions in other regions). The second is that reviewers share a general bias against authors with particular characteristics, regardless of their own personal attributes (e.g. reviewers from institutions in English and non-English speaking countries share a bias against authors from non-English speaking countries). The methodology of the study cannot distinguish between these hypotheses.

There are several ways of detecting generalized bias in peer review scores. These include experimental studies, comparisons between scores when reviewers are blinded to author characteristics and scores when they are not (e.g. 37, 40), analysis of “natural experiments” (as when a journal moves from single blind to double blind review (e.g. 29) and studies that control for the scientific quality of papers, through citations (e.g. 47, 48). Such methods can provide valuable insights. However, experimental methods cannot be applied to operational review systems, “natural experiments” are rare, and the methods that use citations require that the papers in the experimental dataset should have attracted enough to make this into a reasonable measure of quality. The datasets in our own study – one consisting largely of recent papers, the others made up of papers in areas of computer science that attract few citations – did not allow this kind of control. This suggests that different methods of analysis are complementary, and that gaining a more complete picture of bias in peer review requires a plurality of methods.

In none of the datasets, did our study find evidence for a significant interaction between author and reviewer gender. This finding matches results from a previous study which used the interaction between author and reviewer characteristics as a measure of bias ^42^ but contradicts findings from other authors (e.g. 37, 40). The difficulty of automated gender attribution weaken our conclusion for the Frontiers dataset. However, it is plausible that attribution of gender to first names that confused our automated system (mainly Asian names) is equally difficult for reviewers. Furthermore, the errors in the Frontiers dataset did not prevent the detection of a robust difference between the scores obtained by male and female authors, which random errors would tend to obscure. Thus, while we cannot completely exclude gender bias in the Frontiers review process, it is unlikely to have a major practical impact.

Our study also found no evidence for bias in terms of the language and prestige of author institutions and only weak evidence for regional bias. In the case of institutional prestige, it is possible, as in the case of gender, that errors in the input data may have masked some weak effects. However, it is extremely unlikely that they could have hidden effects with a major impact. 

These findings contradict results from previous studies (e.g. 37, 39, 41, 42). Given that the majority of studies showing bias are relatively old, it is possible that changes in social attitudes have reduced or eliminated some of the biases they observed. Furthermore, some of these studies (e.g. 30) measured general biases that were independent of reviewer characteristics, which, as explained earlier, would be invisible to the methodology used here. We suggest, nonetheless, that, at least in the case of gender, general bias is implausible: it is difficult to believe that modern female reviewers are biased against female authors. Furthermore, the only bias we found (regional bias) affected only a very small proportion of the reviews in our datasets. If these findings are correct, the most parsimonious summary of our results is that social bias plays at most a minor role in determining review outcomes. This is especially true for review systems with high acceptance rates where small biases are unlikely to affect final publication decisions. In more selective systems, small biases could have a larger practical impact.

Our study found few significant differences between the scoring patterns of different categories of reviewer. This finding, which held for all three datasets, contrasts with previous studies showing significant differences in scoring practices between male and female reviewers ^43, 49^ and between reviewers from different countries ^49^. However, there have been relatively few studies dedicated to this topic, and even these do not show a major impact of reviewer characteristics on publication decisions ^43^. Taken together, these results suggest that editors should not be over-concerned with the gender, language or institutional affiliation of the reviewers they choose for particular papers, though it could be useful to ensure a good regional balance.

The review systems considered in our study are very different. The majority of papers in the Frontiers dataset came from the life sciences; all the papers in the IEEE datasets were from specialized areas of computer science. Although all three systems in our study, use single blind review, Frontiers adopts a novel interactive review process, which may influence reviewer behavior, even in the first stage of the review process, which is not interactive. The conferences in the IEEE (Spain) and the IEEE (International) datasets use a traditional approach. Authors and reviewers in the Frontiers and the IEEE International datasets come from all over world. The IEEE (Spain) dataset is dominated by authors and reviewers from Southern Europe. Despite these differences, analysis of the three datasets gave similar results. This suggests that the findings of this study could be valid for a broad range of peer review systems. The large size of the datasets used in the analysis (in total 12,943 reviews of 5,753 papers) provides additional evidence of robustness. The main differences between the datasets were in their patterns of regional bias, which are different in each dataset. Unfortunately the many differences between the Frontiers and the IEEE systems make it impossible to untangle the roles of different contributory factors.

Our study does not evaluate the full set of potential biases described in the peer review literature. For instance, we do not consider confirmation bias or alleged reviewer biases in favor of positive results, sophisticated experimental and statistical methodology, or against interdisciplinary studies, replication studies, etc. These are important limitations. A second limitation is that the study makes no attempt to control for the quality of papers, as testified, for example by citations. A third is that the study methodology has so far been tested with just three peer review systems, all applying to scientific papers, and all with relatively high acceptance rates. It is possible that other forms of peer review, such as peer review of grant applications, are subject to different forms of bias.

In conclusion, our study shows that authors' personal characteristics play an important role in determining the scores received in peer review, but finds no evidence that review results are influenced by the personal characteristics of reviewers, and only weak evidence for social bias due to interactions between author and reviewer characteristics. These findings do not rule out generalized bias against authors with specific characteristics or forms of bias not considered in the study.

## Data availability

To protect the identities of authors and reviewers, the source data used for this study has not been made public.

## Supplementary Data

Data files for Walker *et al.* 2015 (V2):
