# Supplementary material for: Personal attributes of authors and reviewers, social bias and the outcomes of peer review: a case study
Source: F1000Res. 2015 Jun 10;4:21. Originally published 2015 Jan 22. [Version 2] doi: 10.12688/f1000research.6012.2 (PMC4648219; doi:10.12688/f1000research.6012.2)
Supplement: Peer review history

# Peer review: Referee response for version 2

**DOI:** 10.5256/f1000research.7075.r8989

**Published:** 2015-06-22

**Authors:** Jigisha Patel

**Affiliations:** 1 Biomed Central Ltd, London, UK

My comments have all been addressed. The revised version presents the authors’ findings in a clearer and more precise way. This serves to highlight the contribution this study makes to the existing literature on bias in peer review. Their efforts to revise to this extent were well worth it.

I don’t have any further comments.

I have read this submission. I believe that I have an appropriate level of expertise to confirm that it is of an acceptable scientific standard.

# Peer review: Referee response for version 2

**DOI:** 10.5256/f1000research.7075.r8988

**Published:** 2015-06-16

**Authors:** Lutz Bornmann

**Affiliations:** 1 Division for Science and Innovation Studies, Administrative Headquarters of the Max Planck Society, Munich, Germany

All reviewers' points have been appropriately addressed.

I have read this submission. I believe that I have an appropriate level of expertise to confirm that it is of an acceptable scientific standard.

# Peer review: Referee response for version 1

**DOI:** 10.5256/f1000research.6434.r7428

**Published:** 2015-02-13

**Authors:** Jigisha Patel

**Affiliations:** 1 Biomed Central Ltd, London, UK

In this paper the authors analyses the relationship between the attributes of authors and reviewers and reviewer outcomes in three datasets, one an innovative peer-review model and two which use traditional peer review. They find no evidence of gender or institutional bias and limited interaction between author and reviewer region.

Although social bias in peer review has been investigated in the past, I think this study is timely and the question of whether this previously found bias still exists with changing social norms and values is interesting. However, there is a need for some clarification of the methods before the significance of the authors’ findings can be determined.

Major revisions

1. The authors are very clear that their study investigates ‘bias’ as defined by the interaction between author *and reviewer* attributes, or bias as a function of reviewer characteristics (ref 29). They make the point that their methodology cannot determine biases that are shared by all reviewers regardless of reviewer characteristics. In the Introduction, the authors switch between describing previous research on reviewer characteristics and previous research on author characteristics. For example, paragraph 2 is predominantly about studies of reviewer characteristics, but ends on author characteristics. Paragraph 3 appears to begins on author characteristics, but then cites studies on reviewer characteristics. It would be much easier for the reader to understand what contribution this study makes to the literature if the authors made a clearer distinction between current  evidence on *reviewer* characteristics (including those studies which looked at both reviewer and author characteristics) and other research on bias, which would include research focused only on author characteristics.
2. As part of the peer review process for Frontiers reviewers can, if they wish, complete the ratings questionnaire shown in Fig 1 and it was these ratings that were used in the study analysis. 

 

I think it would be useful for the authors to clarify the following:

- Do Frontiers reviewers complete the rating independently of each other before the collaborative process? If this is the case, it is not clear to me why individual scores for each paper were averaged for the Frontiers dataset, but apparently not for the other two datasets. If the aim of this study is to investigate the interaction between reviewer attributes and those of authors, wouldn’t averaging the reviewer scores in this way confound this aim?
- Alternatively, if the ratings form is completed by reviewers after the collaborative process, how have the authors accounted for the potential confounding effect of the collaboration?
- Also, if reviewers complete the rating independently of each other, the characteristics of peer review for the Frontiers dataset used in this study are the same as that for the other datasets, i.e. it is single blind peer review. In all three datasets the reviewers are made aware of the authors names, but the authors do not know the reviewers’ – is that correct?
- In the discussion the authors state that the findings of this study could be valid for a broad range of peer review systems. However, this study did not include the interactive component of Frontiers peer review process, or if it did, it is not clear how. All three datasets appear to have used the single blind system of peer review. This statement in the discussion should be rephrased.

Minor

Reference 31 is for a commentary. Can authors should provide the reference for the original Swedish study?

Can the authors provide data on the error rate for their gender assignment process?

I think the authors could provide a more informative title, for example, 'Bias in peer review: the interaction between reviewer and author characteristics.'

Please note, I do not have the expertise to comment on the model and statistical analysis used in this study.

I have read this submission. I believe that I have an appropriate level of expertise to confirm that it is of an acceptable scientific standard, however I have significant reservations, as outlined above.

# Response

**Published:** 2015-05-27

**Authors:** Richard Walker

**Affiliations:** EPF Lausanne, Switzerland

We have now submitted a new version of our paper, which we have revised in the light of your comments, which we found extremely useful and constructive, though they caused us significant extra work. We are particularly grateful for your suggestions concerning the organization of the introduction to the paper, which we have attempted to take on board, and for your points on quality control (which were in the same spirit as comments from Lutz Bornmann. Thanks to these suggestions, we found a number of problems with the data, which had previously passed unnoticed.

- *Comment: The authors are very clear that their study investigates ‘bias’ as defined by the interaction between author and reviewer attributes, or bias as a function of reviewer characteristics (ref 29). They make the point that their methodology cannot determine biases that are shared by all reviewers regardless of reviewer characteristics. In the Introduction, the authors switch between describing previous research on reviewer characteristics and previous research on author characteristics. For example, paragraph 2 is predominantly about studies of reviewer characteristics, but ends on author characteristics. Paragraph 3 appears to begins on author characteristics, but then cites studies on reviewer characteristics. It would be much easier for the reader to understand what contribution this study makes to the literature if the authors made a clearer distinction between current evidence on reviewer characteristics (including those studies which looked at both reviewer and author characteristics) and other research on bias, which would include research focused only on author characteristics.*
  This was an extremely useful suggestion. We have now reorganized our introduction to talk first about effects regarding author characteristics, then to interactions between author and reviewer characteristics and finally to the characteristics of reviewers. We believe the paper gains significantly in clarity from this reorganization.
- *As part of the peer review process for Frontiers reviewers can, if they wish, complete the ratings questionnaire shown in Fig 1 and it was these ratings that were used in the study analysis. Do Frontiers reviewers complete the rating independently of each other before the collaborative process? If this is the case, it is not clear to me why individual scores for each paper were averaged for the Frontiers dataset, but apparently not for the other two datasets. If the aim of this study is to investigate the interaction between reviewer attributes and those of authors, wouldn’t averaging the reviewer scores in this way confound this aim?*
  The ratings questionnaire is filled in in the initial non-interactive part of the review process. We have clarified this in the text.
- *Also, if reviewers complete the rating independently of each other, the characteristics of peer review for the Frontiers dataset used in this study are the same as that for the other datasets, i.e. it is single blind peer review. In all three datasets the reviewers are made aware of the authors names, but the authors do not know the reviewers’ – is that correct?*
  Yes this is correct. We have clarified our description of the Frontiers process to show that it is single-blind.
- *In the discussion the authors state that the findings of this study could be valid for a broad range of peer review systems. However, this study did not include the interactive component of Frontiers peer review process, or if it did, it is not clear how. All three datasets appear to have used the single blind system of peer review. This statement in the discussion should be rephrased*
  The differences between the datasets concern not just the way the review is organized (interactive vs. non-interactive) but also the disciplines covered, and the geographical distribution of authors and reviewers. In the text, we clarify that all three review systems in the study are single blind. However, we hypothesize that reviewers preparing for an interactive process may behave differently from reviewers in a traditional review process.
- *Reference 31 is for a commentary. Can authors should provide the reference for the original Swedish study?*
  As you correctly note, the reference (now reference 30) was published in the form of a commentary. De facto, however, the article represents the first publication of results from an original study, which, to our knowledge was not published elsewhere, prior to the date on which the commentary appeared.
- *Can the authors provide data on the error rate for their gender assignment process?*
  On the basis of random sampling, we estimate errors rates of 7.5%, 0.0% and 5.2% for the Frontiers, IEEE (Spain) and IEEE (International) datasets respectively. We give these figures and discuss their significance in the text.
- *I think the authors could provide a more informative title, for example, 'Bias in peer review: the interaction between reviewer and author characteristics.'*
  We agree with this suggestion and have revised our title accordingly. It now reads, "Personal attributes of authors and reviewers, social bias and the outcomes of peer review: a case study".

# Peer review: Referee response for version 1

**DOI:** 10.5256/f1000research.6434.r7425

**Published:** 2015-02-02

**Authors:** Lutz Bornmann

**Affiliations:** 1 Division for Science and Innovation Studies, Administrative Headquarters of the Max Planck Society, Munich, Germany

Peer review is the most important instrument for assessing scientific research. However, the instrument is not without drawbacks. As the most important weaknesses, a missing reliability, fairness and predictive validity have been seen ^1^. The study of Walker, Barros, Conejo, Neumann and Telefont (2015) deals with the fairness of the peer review process: They investigated “social biases” in the processes of *Frontiers* - an open access publishing house with a novel interactive peer review process, and two peer review processes from Spanish and international computer science conferences.

The study is very interesting. I recommend that the authors revise the manuscript according to the following points:

1. On page 5, Walker, *et al*. describe the process of normalizing authors’ and reviewers’ names. Here, Walker, *et al.* should ensure that the names are completely cleaned: the same author and the same institutional unit should receive the same name. A general problem of this kind of data from peer review processes is that they are not cleaned and contain several name variants.
2. Walker, *et al. * present their results with the reporting of statistical significance information. I recommend that not only the statistical, but also the practical significance of the results (effect sizes) should be reported. ^2^^,^^3^^,^^4^
3. In the Discussion section the following major limitation of the study should be mentioned: The quality of the papers was not controlled. For example, Bornmann and Daniel (2009) ^5^ and Bornmann and Daniel (2010) ^6^ investigated the peer review processes of the *Angewandte Chemie – International Edition* and *Atmospheric Chemistry and Physics*. They considered citations for the single papers as a proxy for quality. Although citations measure only one part of quality (namely impact), it is more favorable to consider them than doing not. When examining the association of bias variables and peer review outcomes without controlling quality it is impossible to establish unambiguously whether a particular group of papers receives more favorable recommendations or decisions due to these variables, or if the more favorable recommendations and decisions are simply a consequence of the papers’ scientific quality.

I have read this submission. I believe that I have an appropriate level of expertise to confirm that it is of an acceptable scientific standard, however I have significant reservations, as outlined above.

## References

[1] BornmannL: Scientific peer review. *Annual Review of Information Science and Technology*.2011;45(1) : 10.1002/aris.2011.1440450112 197-245 10.1002/aris.2011.1440450112

[2] CohenJ: Statistical power analysis for the behavioral sciences (2nd Ed). 1988;Lawrence Erlbaum Associates Hillsdale, NJ, USA: Reference source

[3] WilliamsR: Using the margins command to estimate and interpret adjusted predictions and marginal effects. *The Stata Journal*.2012;12(2) :308-311 Reference source

[4] WilliamsRBornmannL: The substantive and practical significance of citation impact differences between institutions: guidelines for the analysis of percentiles using effect sizes and confidence intervals. *In Y. Ding, R. Rousseau & D. Wolfram (eds.), Measuring scholarly impact: methods and practice*.2014;Springer, Heidelberg, Germany:258-281 Reference source

[5] BornmannLDanielH-D: Reviewer and editor biases in journal peer review: an investigation of manuscript refereeing at Angewandte Chemie International Edition. *Research Evaluation*.2009;18(4) : 10.3152/095820209X477520 262-272 10.3152/095820209X477520

[6] BornmannLDanielH: Do Author-Suggested Reviewers Rate Submissions More Favorably than Editor-Suggested Reviewers? A Study on Atmospheric Chemistry and Physics. *Plos One*.2010;5(10) : 10.1371/journal.pone.0013345 10.1371/journal.pone.0013345 PMC295479520976226

# Response

**Published:** 2015-02-11

**Authors:** Richard Walker

**Affiliations:** EPF Lausanne, Switzerland

I think these comments are very relevant and we will take them into account in the next version of our paper. As concerns the specific points you raise:

1. In our study, authors' and reviewers' names are relevant for gender assignment. Institution names are critical for assignment of language, region, and institutional prestige to author and reviewer institutions. Informal checks on our cleaning process suggest that it does not introduce substantial errors into our analysis. In the next version of our paper we will introduce more formal checks.
2. We agree with the reviewer on this point. The next version of our paper will include estimates of the practical significance of our results. 
3. We completely agree with the reviewer that some differences in scores may be due to differences in scientific quality and tried to make this point in our text. In the next version, we will attempt to clarify this issue.

# Response

**Published:** 2015-05-27

**Authors:** Richard Walker

**Affiliations:** EPF Lausanne, Switzerland

Thank you for your comments, which we found extremely useful and constructive. Particularly useful was your suggestion that we should include effect sizes in our analysis. This suggestion has been implemented in this revised version of our paper. We would also like to thank you for your suggestions regarding quality control, complementing similar suggestions from Jigisha Patel. Thanks to these suggestions, we found a number of problems with the data, which had previously passed unnoticed. All these changes have been incorporated in the new version of the paper which we have just submitted.

- *On page 5, Walker, et al. describe the process of normalizing authors’ and reviewers’ names. Here, Walker, et al. should ensure that the names are completely cleaned: the same author and the same institutional unit should receive the same name. A general problem of this kind of data from peer review processes is that they are not cleaned and contain several name variants.*
  Thank you this comment, which, together with comments in a similar vein from Jigisha Patel led us to conduct a thorough review of our data. The review found that errors in the normalization of author, reviewer and institution names and missing values led to down-stream errors in automated gender assignment and in the assignment of university rankings. In the case of the university rankings we were able to correct a number of errors. In the case of the gender assignment, this would have been too onerous to be possible. Instead, as suggested by Jigisha Patel, we used sampling to check the error rates in our data, which we report in our text, together with a discussion of their significance.
- *Walker, et al.  present their results with the reporting of statistical significance information. I recommend that not only the statistical, but also the practical significance of the results (effect sizes) should be reported. ^2,3,4^*
  Thank you for this suggestion, which we have implemented in the revised version of our manuscript. We began by calculating Cohen's d for all our data. In the results and the discussion sections, we analyse the practical significance of the effects observed both in terms of the number of reviews concerned (which was always small) and the effects on publication decisions. In the cases we study, this was not large. However, in more selective review systems it could be larger.
- *In the Discussion section the following major limitation of the study should be mentioned: The quality of the papers was not controlled. For example, Bornmann and Daniel (2009) ^5^ and Bornmann and Daniel (2010) ^6^ investigated the peer review processes of the Angewandte Chemie – International Edition and Atmospheric Chemistry and Physics. They considered citations for the single papers as a proxy for quality. Although citations measure only one part of quality (namely impact), it is more favourable to consider them than doing not. When examining the association of bias variables and peer review outcomes without controlling quality it is impossible to establish unambiguously whether a particular group of papers receives more favourable recommendations or decisions due to these variables, or if the more favourable recommendations and decisions are simply a consequence of the papers’ scientific quality*
  We now make explicit reference to this limitation in our text. We go on to explain that most of the papers in our datasets have very few citations. This means, that in our case, we are unable to use citations as a proxy for quality.
